# Assessment of Stress Caused by Environmental Changes for Improving the Welfare of Laboratory Beagle Dogs

**DOI:** 10.3390/ani13061095

**Published:** 2023-03-19

**Authors:** Gwang-Hoon Lee, Woori Jo, Tae-Ku Kang, Taeho Oh, KilSoo Kim

**Affiliations:** 1 Preclinical Research Center, Daegu-Gyeongbuk Medical Innovation Foundation, Daegu 41061, Republic of Korea; 2 Department of Veterinary Internal Medicine, College of Veterinary Medicine, Kyungpook National University, Daegu 41566, Republic of Korea; 3 Department of Veterinary Toxicology, College of Veterinary Medicine, Kyungpook National University, Daegu 41566, Republic of Korea

**Keywords:** animal welfare, cortisol, dog, environmental enrichment, stress

## Abstract

**Simple Summary:**

Stress is an inevitable element in the course of life that must be accepted, but efforts to minimize it are necessary. In particular, since captive animals in animal testing centers can experience relatively high levels of stress, efforts should be made to alleviate their stress. The aim of this study was to find a suitable environment that can reduce the stress of captive dogs. We conducted a scientific evaluation of the stress caused by environmental changes in dogs. According to the present results, social housing and environmental enrichment reduce dogs’ stress.

**Abstract:**

Animal stress is influenced by environmental factors, yet only a few studies have evaluated the effects of environmental stress on captive dogs. This study aimed to evaluate the effects of environmental and social enrichment on the stress levels of captive dogs housed in a lab. We assessed stress levels in eight Beagle dogs by measuring their body weight, cortisol levels, a stress hormone, the alkaline phosphatase activity in serum, the number of steps per hour, as well as clinical sign observations in a changed environment for 6 weeks. Four dogs assigned to a control group were raised alone in a relatively narrow place without toys; four dogs assigned to an experimental group were raised together in a relatively large place with toys. The body weight of the control group remained unchanged, while that of the experimental group decreased. Cortisol levels in the control group increased throughout, whereas those in the experimental group increased for up to 2 weeks and decreased thereafter. Consequently, cortisol levels in the experimental group significantly decreased compared to the control group at 6 weeks (*p* = 0.048). Fighting was observed among the dogs in the experimental group at 3 weeks; thus, one dog was separated from the group. The number of steps per hour was more than twice as high in the experimental than in the control group. Thereby, we determined that social housing, with appropriate companions and environmental enrichment materials, can reduce stress levels in captive dogs more efficiently than in single housing without such materials. Our study provides useful insights for captive animal organizations, such as kenneled dogs’ management, to improve animal welfare.

## 1. Introduction

Stress can be defined as the process of changing the physical and psychological state to protect the body against external threats and attacks, as well as non-specific biological reactions occurring in the body in response to various injuries and stimuli applied to the living body [1,2,3]. Stress can be categorized as either eustress or distress [4]. Eustress, called positive stress, can result in increased happiness or motivation when encountered. Conversely, distress is elicited by a negative or unpleasant stressor and is commonly associated with the stress response [4]. Stress is commonly used to refer to distress [5].

Animals, including companion animals and captive animals, are known to have increased stress levels due to noise, restriction in kennels, and the presence or lack of companions [6,7]. In particular, captive animals in animal research institutions, animal shelters, or zoos require strict stress management, unlike wild animals or companion animals, because they spend their entire lives in confined spaces [8,9,10]. A previous study reported that the most stressful thing for captive animals is not being able to escape the potential stresses of the artificially created environment, so these stresses should be assessed, and improvements or changes should be made as they are essential for the welfare of captive animals [6].

Dogs are suitable species to be bred as companion animals because they are well known to form bonds with humans [11]. However, in the non-clinical stage for entering clinical trials, animal studies using dogs are often conducted. The efficacy evaluation of new drug candidates and the development of medical devices are mainly conducted using rodents because of their short life span, low cost, and easy handling [12,13,14]. Non-clinical studies using rodents have provided important mechanistic insights, however, their application for non-clinical evaluation is limited due to their anatomical and pathophysiological differences from humans [15,16]. In contrast, non-rodent laboratory animals such as dogs are relatively similar to humans and therefore have higher values in non-clinical studies [17]. Dogs are valuable non-clinical research animals because of the wide range of research subjects and their relative availability among non-rodent laboratory animals [18,19]. Since safety evaluations require testing on one species of rodents, such as mice or rats, and one species of non-rodents, such as rabbits, dogs, pigs, dogs, or non-human primates, animal experiments on dogs are routinely conducted [20]. However, confinement to limited space, social isolation, exposure to unfamiliar surroundings, and prolonged stays in kennels are all potential factors that can lead to reduced welfare for dogs in shelters [21]. Dogs that are taken to animal shelters often display both physical and behavioral indications of being under stress [22]. The cortisol levels in dogs living in a shelter environment during the initial three-day period are nearly three times higher than the levels in household dogs [23]. Laboratory studies have investigated the physiological and behavioral impacts of stress, how to alleviate it, as well as the psychological and behavioral consequences of unchecked stress reactions, with several of the stressors used in these studies closely resembling the conditions experienced by dogs that are kept in animal shelters [24,25,26]. Although human interaction can reduce the stress levels of captive dogs, dogs in animal research facilities and animal shelters are inevitably exposed to long periods of time without humans, contrary to companion animals [23]. It is important to develop strategies that can help alleviate their stress when they are in a confined environment and without the presence of humans.

In particular, the provision of toys as environmental enrichment materials was commonly recognized as essential for reducing stress levels and enhancing the welfare of captive animals [27]. However, there is conflicting evidence regarding the efficacy of toys in improving animal welfare. While some studies report the positive effects of toys in reducing abnormal behaviors and increasing activity levels [25,26], other studies suggest that toys have no significant impact on animal behavior or well-being [28,29].

The absence of social contact with conspecifics is known to induce stress in dogs [30]. It leads to negative behavioral changes, such as decreased activity levels and increased stereotypic behavior [29]. Therefore, the social housing of dogs is commonly pursued in animal shelters and animal research facilities [21,31].

Few studies that have evaluated animal stress levels are mainly limited to rodents in animal research facilities, which are not as similar to humans compared to non-rodent animals, such as dogs [32,33].

Physical activity and stress interact with each other. Exposure to stress can hinder achieving a healthy level of physical activity, while physical activity can lead to positive physiological changes in mental health, such as improving self-esteem and reducing stress and anxiety levels [34,35]. Nevertheless, it is undeniable that engaging in moderate exercise can provide healthy advantages [36,37]. Therefore, through the quantification of activity levels, body weight, and body condition score as methods for assessing relative fatness related to animals’ health conditions [38], it is possible to discern the health status of dogs and potentially establish correlations with stress.

The activation of the hypothalamic-pituitary-adrenal (HPA) axis in response to stress can lead to increased levels of cortisol and alkaline phosphatase (ALP) activity in serum [39,40].

In the present study, to evaluate the stress of single housing without companions and environmental enrichment (control group) as well as and social housing with companions and environmental enrichment (E.E group), changes in cortisol concentration, alkaline phosphatase activity, blood tests, weight, and the number of steps per hour were measured. We hypothesized that environmental enrichment within a large space might reduce stress levels and increase the activity of captive dogs.

## 2. Materials and Methods

### 2.1. Animals

Eight unneutered male Beagle dogs aged from 18 to 21 months (ORIENT BIO, Jeongeup, Korea), weighing 12–14 kg, were used in this experiment after having been vaccinated with canine distemper virus, canine adenovirus (infectious canine hepatitis), canine parvovirus, canine parainfluenza virus, and *Leptospira* spp. Since young and immature dogs show a high ALP activity due to bone growth, dogs aged 18 to 21 months with skeletal maturity were used [39,41]. The dogs were raised at the Preclinical Research Center (PRC), Daegu-Gyeongbuk Medical Innovation Foundation (K-MEDI hub), accredited by AAALAC International (#001796) in accordance with the guide for the Care and Use of Laboratory Animals 8th edition, NRC (2010). The dogs were fed a limited amount (300 g/dog/day) of a laboratory dog diet (Production Number 38070, Cargill Agri Purina Incorporated, SeongNam, Republic of Korea) with free access to water purified by a microfiltration system and reverse osmosis process. Dogs were placed in stainless steel cages (size of a cage: 1.12 m wide × 1.62 m deep × 1.85 m high) at room temperature 22 ± 1 ℃, 50 ± 10% humidity, 1.8–4.2 mmAq positive room pressure, and a ventilation cycle of 10–20 times/h.

### 2.2. Housing Environment

**Figure 1:**
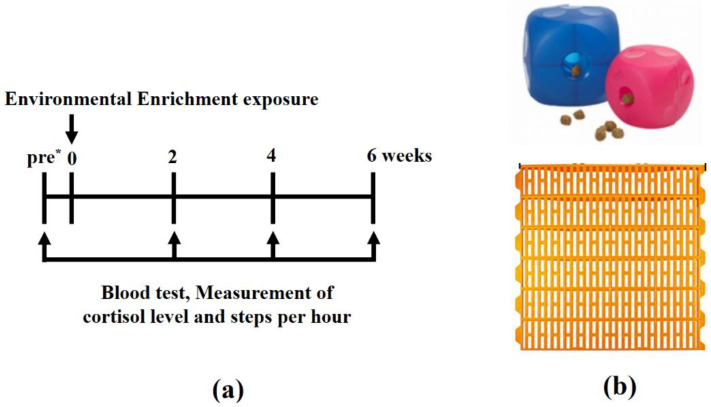
(**a**) Diagrammatic representation of the experimental protocol, * pre: before environmental enrichment exposure; (**b**) environmental enrichment materials (Buster soft cube, top; soft plastic floorboard, bottom).

After an acclimation period of two weeks, the eight dogs were divided into two groups at random, either assigned to the control group or the environmental enrichment (E.E) group in the PRC, K-MEDI hub. As a control group, four dogs were provided with an environment in which they were raised in n 1.814 m^2^ (size of a cage: 1.12 m wide × 1.62 m deep × 1.85 m high) cage without toys on the stainless steel floor. On the other hand, the E.E group of four dogs was provided with an environment in which they were raised in a 7.258 m^2^ (size of a cage: 4.48 m wide × 1.62 m deep × 1.85 m high) cage constructed by connecting four cages with a soft plastic floorboard (TAEWOO, Seongju, Korea). The four dogs were provided with four toys (Buster Soft Cube, Product number 27452, dimension 12.7 × 12.7 × 12.7 cm, Kruuse, Langeskov, Denmark) as environmental engineering in the connected cage (Figure 1). The toys were exchanged daily for a cleaned one and were always present in the cage. Food was inserted into the toy every day, and when the dog rolled the toy, the food came out little by little.

### 2.3. Observation of Clinical Signs and Body Weight with Body Condition Score Measurement

Clinical signs were observed by visual inspection every day and by palpation once a week. The body weight and the body condition score (BCS) were measured once a week. If a fight due to social housing was found in the E.E group, the dog was separated from the rest. The BCS is a method for assessing relative fatness related to the animals’ health conditions [38]. The BCS was measured by the criteria of the World Small Animal Veterinary Association [38].

### 2.4. Cortisol Concentration in Serum Measurement and Blood Tests

To determine the cortisol concentration in the serum and to perform other blood tests, blood was sampled from the cephalic vein at 2-week intervals, including before the environmental change of placing them into the cages, for a total of 6 weeks. Sampling was performed once only between 17:00 and 18:00, taking into account the end of breeding management and other researchers’ entry and exit. Blood sampling was conducted in less than 2 min to minimize the stress caused by blood sampling because the handling of animals can induce stress [42]. The blood was collected in ethylenediaminetetraacetic acid (EDTA) tubes and serum-separating tubes (SST), followed by centrifugation at 1500 g for 10 min at 4 °C for collecting the serum after 30 min of clotting time. The cortisol concentration in the serum was measured directly using an immunoassay with the immulite 2000 xpi (Siemens, Eschborn, Germany) with a limit of detection at 0.2 µg/dL. The hematology (white blood cells, red blood cells, hemoglobin, hematocrit, mean corpuscular volume, mean corpuscular hemoglobin, mean corpuscular hemoglobin concentration in blood, red blood cell distribution width, platelets, and WBC differential count) was analyzed using a hematology system with autoslide (ADVIA 2120i, Siemens, WA, USA). The biochemistry (alkaline phosphatase, aspartate aminotransferase, albumin, alanine aminotransferase, total bilirubin, triglyceride, blood urea nitrogen, calcium, creatinine, inorganic phosphorus, glucose, sodium, total cholesterol, potassium, total protein, and chloride) was analyzed using a clinical chemistry analyzer (TBA-120FR, Toshiba, Tokyo, Japan).

### 2.5. Feed Consumption Measurement

Three hundred grams of feed per dog was supplied daily, and the remaining amount was checked after 24 h to measure daily feed intake. However, in the case of the E.E group, it was not possible to confirm which dog consumed the feed due to social housing; therefore, the average feed intake of both groups was calculated and compared.

### 2.6. Steps per Hour Measurement

The number of steps per hour was measured using a canine activity tracker (FitBark, Kansas City, MO, USA). Each dog was fitted to the activity tracker on their neck on a light leash, and the number of steps per hour was measured for 3 hours in a day at 2-week intervals, including before the environmental change of placing them into the cages, for a total of 6 weeks.

### 2.7. Statistical Analyses

All statistical tests were performed with. the GraphPad Prism 8.0 statistical software (GraphPad Software, La jolla, CA, USA). All data were expressed as the mean value ± the standard deviation value (S.D). Multiple t-tests were used to analyze all data between groups after checking the normality test using the Shapiro–Wilk test. The Kruskal-Wallis tests were used to analyze all data within the groups after confirming that they did not pass the Shapiro–Wilk test of normality. Values of *p* < 0.05 were considered statistically significant.

## 3. Results

### 3.1. Clinical Signs and Body Weights with Body Condition Scores

**Figure 2:**
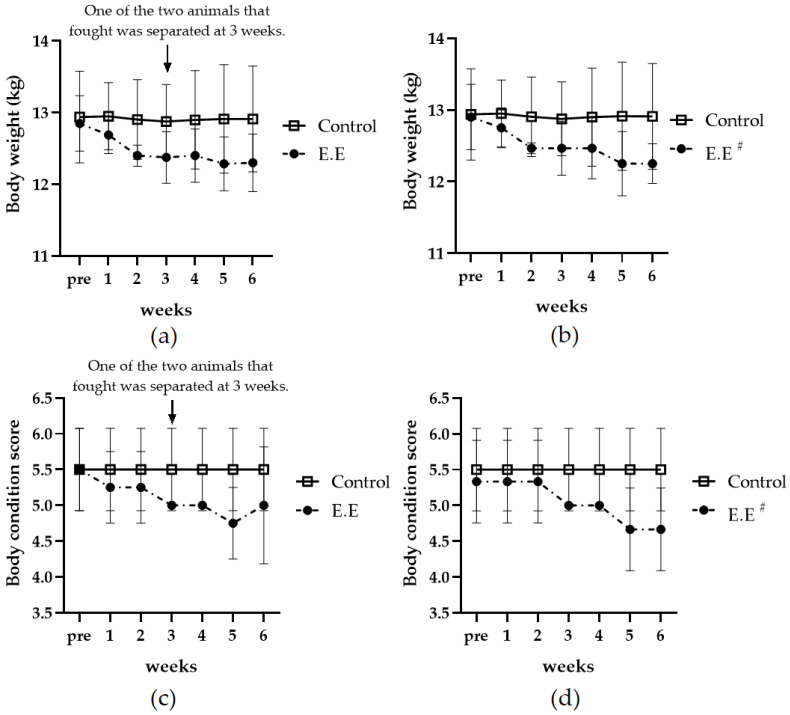
Change in body weights and body condition score (BCSs) at 1-week intervals, including before the environmental change of placing them into the cages (pre) for a total of 6 weeks. Change in body weight when the separated fighting dog was (**a**) included and (**b**) not included. Change in body condition scores when the separated fighting dog was (**c**) included and (**d**) not included. E.E: Environmental enrichment group. ^#^ Except for the one dog that fought.

In the E.E group, fighting between two dogs was observed, and one of them was separated and reared alone in the same cage as the one belonging to the control group at 3 weeks, however, toys were provided. No statistically significant difference could be confirmed in the body weights and the body condition scores (BCSs) between the two groups during the experimental period (*p*-value: 0.657 in 1 week, 0.622 in 2 weeks, 0.657 in 3 weeks, 0.657 in 4 weeks, 0.657 in 5 weeks, and 0.657 in 6 weeks). There were also no significant differences within the groups. However, the control group showed little changes in weight with a BCS of 5.50 ± 0.58 during the experimental period, whereas the E.E group had weight loss compared to the pre- E.E group, with a weight loss of 0.56 ± 0.35 kg with a BCS of 4.75 ± 0.50 at 5 weeks, and a weight loss of 0.55 ± 0.40 kg with a BCS of 5.00 ± 0.82 at 6 weeks when the fighting dogs were included. In addition, the E.E group showed an average weight loss of 0.65 ± 0.38% with a BCS of 4.67 ± 0.58 at 5 weeks and an average weight loss of 0.70 ± 0.33 kg with a BCS of 4.67 ± 0.58 at 6 weeks when the fighting dogs were not included (Figure 2). The separated dog lost weight by week 3, after which the weight increased. Moreover, the BCS was 6 at pre, but it decreased to 5 from the 1st to the 5th week, and then it increased to 6 at the 6th week (Figure S1).

### 3.2. Steps per Hour Measurement

**Figure 3:**
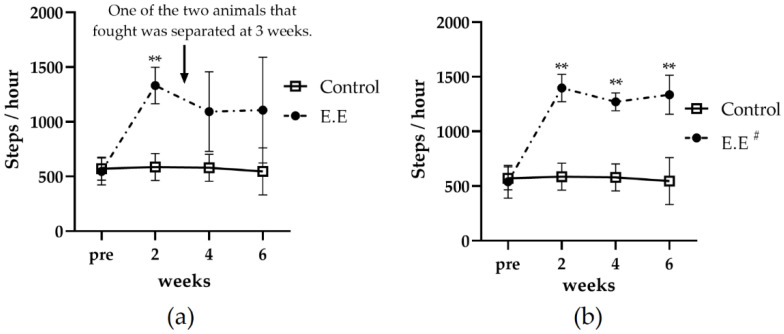
Steps per hour measurement at 2-week intervals, including before the environmental change of placing them into the cages (pre) for a total of 6 weeks when the separated fighting dog was (**a**) included and (**b**) not included. E.E: Environmental enrichment group. ** *p* < 0.01 vs. control group. ^#^ Except for the one dog that fought.

In the control group, no significant changes were determined over time. In the E.E group, the number of steps per hour was significantly higher than that in the control group at 2 weeks when the separated fighting dog was included (*p*-value: 0.001 in 2 weeks, 0.106 in 4 weeks, and 0.151 in 6 weeks), and significantly higher than that in the control group at 2, 4, and 6 weeks when the separated fighting dog was not included (*p* = 0.01 in 2 weeks, 0.001 in 4 weeks, and 0.007 in 6 weeks) (Figure 3). There were also no significant differences within the groups when the separated fighting dog was included or not included. In the case of the separated dog, the number of steps per hour increased at 2 weeks compared to pre, and it decreased at 4 and 6 weeks compared to 2 weeks (Figure S2).

### 3.3. Feed Consumption

**Figure 4:**
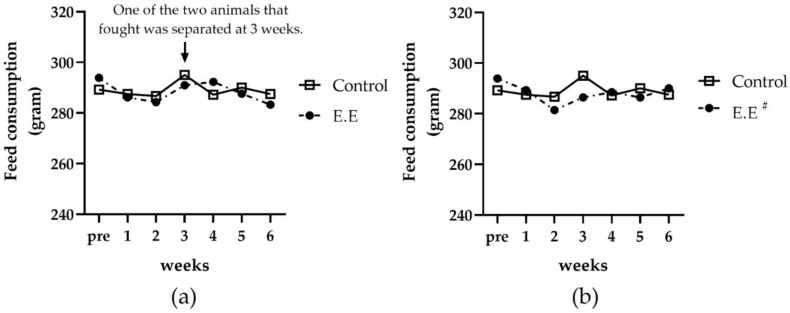
Feed consumption at 1-week intervals, including before environmental change of placing them into the cages (pre) for a total of 6 weeks when the separated fighting dog was (**a**) included and (**b**) not included. ^#^ Except for the one dog that fought. E.E: Environmental enrichment group.

The average feed consumption per individual was between 280 g and 293 g in all groups during the experimental period. Therefore, there was no change greater than 4.64% (293–280) in the average feed intake over the study period (Figure 4). In the case of the separated dog, the feed consumption was between 277 g and 300 g (Figure S3).

### 3.4. Cortisol Concentration in Serum

The average cortisol concentration in the collected serum increased in the control group during the experimental period without any significant differences (pre: 0.66 ± 0.32, 2 weeks: 0.93 ± 0.54, 4 weeks: 1.27 ± 0.53, and 6 weeks: 1.38 ± 0.21 µg/dL). In the E.E group, the cortisol value at 2 weeks was statistically elevated compared to the cortisol value at the pre when the separated fighting individual was included (*p* = 0.031). There were no significant differences within the E.E group when the separated fighting individual was not included.

**Figure 5:**
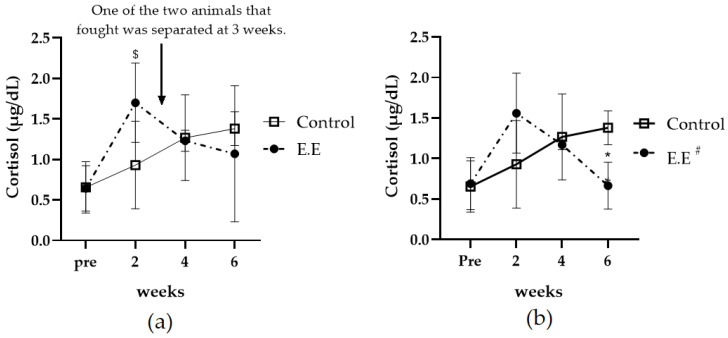
Change in cortisol concentration in collected serum at 2-week intervals, including before environmental change of placing them into the cages (pre) for a total of 6 weeks when the fighting dog was (**a**) included and (**b**) not included. ^#^ Except for one dog that fought. E.E: Environmental enrichment group. ^$^ *p* < 0.05 vs. pre, * *p* < 0.05 vs. control group.

When the separated fighting individual was included, there was no significant difference between two groups (cortisol value: pre: 0.64 ± 0.28, 2 weeks: 1.70 ± 0.49, 4 weeks: 1.23 ± 0.13, and 6 weeks: 1.07 ± 0.84 µg/dL; *p*-value: 0.281 in 2 weeks, 0.989 in 4 weeks, and 0.875 in 6 weeks). When the separated fighting individual was not included, the cortisol concentration was significantly lower at 6 weeks in the E.E group (cortisol value: pre: 0.69 ± 0.32, 2 weeks: 1.56 ± 0.49, 4 weeks: 1.17 ± 0.06, and 6 weeks: 0.67 ± 0.29 µg/dL; *p*-value: 0.438 in 2 weeks, 0.947 in 4 weeks, and 0.048 in 6 weeks) (Figure 5). In the case of the separated dog, compared to the pre, the cortisol levels increased at 2 weeks, decreased at 4 weeks, and then increased again at 6 weeks (Figure S4).

### 3.5. Alkaline Phosphatase Activity

**Figure 6:**
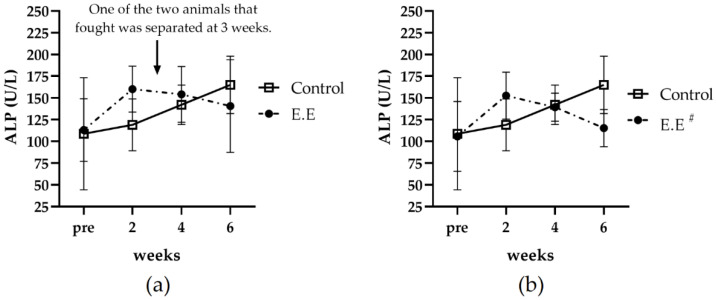
Change in ALP activity at 2-week intervals, including before environmental change of placing them into the cages (pre) for a total of 6 weeks when the fighting dog was (**a**) included and (**b**) not included. E.E: Environmental enrichment group. ^#^ Except for one dog that fought.

No statistically significant difference could be confirmed between the two groups in terms of the alkaline phosphatase (ALP) activity at any time interval when the separated fighting individual was included (*p*-value: 0.301 in 2 weeks, 0.846 in 4 weeks, and 0.846 in 6 weeks) and when the separated fighting individual was not included (*p*-value: 0.460 in 2 weeks, 0.983 in 4 weeks, and 0.267 in 6 weeks). However, changes in the average ALP activity were similar to changes in cortisol levels. In the control group, the ALP activity continued to increase throughout the test period. In the E.E group, the average ALP activity increased for up to 2 weeks and then decreased without a significant difference (Figure 6). In the case of the separated dog, the ALP activity increased over time (Figure S5).

## 4. Discussion

Stress refers to the state of physical and psychological tension that an individual feels when faced with a difficult situation to adapt to, and it affects various organs due to physiological changes [43,44,45]. We hypothesized that stress levels would decrease when environmental enrichment and appropriate companions were provided. Previous studies have also studied the benefit of environmental enrichment for stress reduction. Hunt et al. demonstrated that environmental enrichment increases relaxation behavior and decreases alert and stress behaviors in dogs [46]. Albanese et al. reported that environmental enrichment increases their positive behavior and reduces the fecal cortisol metabolite in the study of non-human primates, which are another captive animal species. Additionally, their study emphasized that welfare programs considering animal species are important [47]. In terms of the body weight and body condition score (BCS), the E.E group showed an average decrease. However, the effects of stress on weight are conflicting. When stressed, humans consume more sugary, fatty foods and more energy-dense meals, which can lead to obesity [48,49,50]. However, previous studies have revealed that stress causes weight loss along with poor feed intake [51,52,53]. Another study additionally found that stress induces anorexia nervosa and a reduction of appetite and food consumption in dogs, and dogs with positive affective states had a stronger tendency to seek food than dogs with negative affective states [54,55].

In the present study, while the weight of the control group did not change, the weight of the E.E group decreased over time, but we did not judge this result to be an effect of stress. Since all of the dogs used in this study were between 18 and 21 months of age, their skeletal maturity had already been completed [41]. In addition, the BCS was 5.50 ± 0.58 in both pre-experimental groups, however, the BCS decreased after 1 week. For dogs, a BCS of 4 or 5 is ideal, and a BCS > 5 of 9 is considered overweight [56]. Both groups were in a slightly overweight state pre-experiment (BCS: 5.50 ± 0.58); however, only the E.E group recovered to an ideal BCS of 5.00 ± 0.82 when the fighting dogs were included, and a BCS of 4.67 ± 0.58 when the fighting dogs were not included at 6 weeks.

This result is supported by the number of steps per hour and the daily feed intake. The relatively large floor area and companions made active exercise possible, and the number of steps per hour was measured at more than twice that of the control group. However, no significant difference could be confirmed in the feed intake between the two groups during any of the experimental periods. Therefore, we judged that even if the E.E group lost weight, it was not due to stress but a normal physiological response to vigorous exercise, as demonstrated in previous studies [57,58].

Activities such as walking have lots of benefits for dogs in disease prevention, as well as for their mental and social health [59]. However, the heightened levels of activity in dogs residing in animal shelters may be attributed to the challenging conditions of the environment, including exposure to various stimuli such as sights, sounds, and odors that make it difficult for them to rest [60]. Given that marked increases and decreases in activity are both recognized indicators of stress, the level of activity can be considered a sign of stress and a coping mechanism employed by dogs to manage their stress levels [61]. In addition, repetitive locomotor stereotypies such as pacing, circling, and spinning, which result in a high number of steps, are commonly observed in confined environments, such as animal research facilities and rescue shelters, however, this does not necessarily lead to a reduction in stress [62]. Thus, although our study found that the increased physical activity in the E.E group had a positive effect on their health by recovering to an ideal BCS, it is inconclusive whether this intervention led to a reduction in cortisol levels. In puppies, a stress-hyporesponsive period (SHRP) exists during which endocrine responses, including cortisol secretion, are suppressed. The SHRP ends at approximately 5 weeks of age and coincides with the termination of lactation [63]. Dogs that are too old exhibit a higher concentration of cortisol, likely due to a decline in stress resistance [64]. We determined that the age range of 18 to 21 months in dogs would be suitable for conducting experiments related to stress. Nevertheless, longitudinal investigations on stress levels in young puppies are of great value since mild stress experienced during this early stage of development can accelerate maturation, enhance problem-solving abilities, and increase social confidence later in life [65,66].

Cortisol, which is an adrenocorticotropic hormone (ACTH)-dependent glycosteroid secreted from the adrenal cortex, is a hormone that is indispensable for maintaining life [67]. Cortisol is the most important hormone for maintaining the homeostasis of the body and in response to stress [40,68]. Stress stimulates the release of the corticotropin-releasing hormone from the hypothalamus, which elevates the secretion of ACTH from the pituitary gland. The ACTH increases the secretion of cortisol from the adrenal cortex, increasing the concentration of cortisol in the blood. The hypothalamic-pituitary-adrenal (HPA) axis is the major pathway for cortisol secretion [69,70]. Cortisol secretion can be induced in the adrenal cortex by various stress factors, such as fatigue, irritability, sleep insufficiency, high-intensity exercise, panic, suffering, or hunger [67,71]. In particular, dogs can experience stress from separation anxiety, changes in routine, loud noises, fear of new situations or unfamiliar people, medical conditions, lack of socialization, and overstimulation [72,73,74]. The secreted cortisol affects muscles, the liver, and fatty tissue to provide energy for the subject to resist stress [75]. Therefore, many studies have evaluated stress in animals under various conditions, such as heat stress or water restriction, both of which strongly induce stress, with cortisol increase as the major factor in stress evaluation [76].

The average cortisol concentration in the collected serum of the control group continued to increase during the experimental period without any significant difference, while the average cortisol concentration of the E.E group significantly increased at 2 weeks compared to the pre and then decreased thereafter without any significant difference. The routine life and single housing of the control group probably caused stress, as recorded in a previous study, and we determined that the stress became more severe over time. The reason that the average cortisol level in the E.E group increased by 1.83 times, compared to the control group at 2 weeks when the fighting dog was included, was probably because of the presence of an inappropriate companion. It may have been time to adapt to a new group until 2 weeks considering the possible competition for enrichment with the inappropriate companion. In our study, dogs consumed most of the provided feed, and the toys that were provided as environmental enrichment materials contained feed. It should be noted that excessive environmental enrichment, particularly when it involves food-based stimuli, may have unintended consequences such as increased competition and altered behavior, as demonstrated in a previous study [77].

The cortisol level significantly decreased after that companion was removed compared to the control group, so it was determined that the stress decreased rapidly due to that removal. The cortisol level in the E.E. group was lower than in the control group at 6 weeks.

ALP can be used as a stress factor because it is released after the ACTH stimulates the adrenocortical cells [39,51]. Since both cortisol level and ALP activity increase by the stimulus of the ACTH, we noticed a similar trend of the two factors, as found in previous research. Zimmerman et al. reported that the cortisol level was correlated with the ALP activity after the ACTH stimulation in dogs [78].

The separated dog lost weight by week 3 but gained weight after the separation. In addition, although the number of steps per hour increased in the 2nd week, the average number of steps per hour decreased similarly to the control group in the 4th and 6th weeks after separation. This explains the elastic changes in the body weight and the BCS with cage size. In addition, the cortisol level may have increased by 2 weeks due to the conflict with another dog, then decreased by 4 weeks after separation and eliminating the conflict, and then increased by 6 weeks because of the single housing routine.

Respecting the natural behaviors of animals plays a major role in animal welfare [79]. We provided the E.E group with a relatively large area, companions, toys for foraging, and a soft floor to encourage their natural behavior. According to our results, we can support the hypothesis that the environment in the E.E. group reduced stress levels in dogs.

A limitation of our study is that several factors, including social housing and environmental enrichment, were applied at once. The small sample size and having only one dog breed (Beagle) are also limitations of this study because captive animals in the zoo or animal shelters consist of various breeds, although animal research facilities almost always use Beagle dogs. In future studies, it will be necessary to evaluate which factors reduce stress levels by setting up multiple groups and applying various factors individually, and various breeds with larger sample sizes should be included to improve the reliability of the study. Additionally, it is necessary to require non-invasive methods, such as behavioral evaluation and cortisol analysis of feces, rather than blood collection by referring to a previous study [47].

Nevertheless, our study proved that the provision of environmental enrichment with appropriate companions reduces stress levels in dogs. This is based on the results that the E.E group had lower cortisol levels and ALP activity compared to the control group and that a wide environment in which exercise was possible helped the physical health of the animals by maintaining an ideal BCS. Our results suggest appropriate improvements to the breeding guidelines for captive dog management in facilities such as animal testing facilities.

## 5. Conclusions

We evaluated the effects of environmental stress on dogs living in a restricted area in an animal research institute. Our experimental results suggested that rearing dogs in a large space with social housing, appropriate companions, and environmental enrichment reduces animal stress more than rearing dogs in a small space in single housing without environmental enrichment. However, it is important to consider that certain enrichment materials, such as toys, may have caused social conflicts among dogs. Furthermore, excessive movements in social housing may interfere with rest and their overall well-being, highlighting the need to carefully evaluate the effectiveness of environmental enrichments in reducing stress levels. Our scientific experiment results may suggest a way to improve the welfare of captive dogs.

## Data Availability

The data presented in this study are available upon request from the corresponding author.

## Supplementary Materials

The following supporting information can be downloaded at: https://www.mdpi.com/article/10.3390/ani13061095/s1, Figure S1: Body weights and body condition scores (BCS) in separated dog; Figure S2: Feed consumption in separated dog; Figure S3: Steps per hour in separated dog; Figure S4: Cortisol level in separated dog; Figure S5: Alkaline phosphatase (ALP) activity in separated dog.
