# Supplementary material for: Assessment of Stress Caused by Environmental Changes for Improving the Welfare of Laboratory Beagle Dogs
Source: Animals (Basel). 2023 Mar 19;13(6):1095. doi: 10.3390/ani13061095 (PMC10044678; doi:10.3390/ani13061095)
Supplement: Supplementary file 1 [file animals-13-01095-s001.zip › animals-2257291-supplementary.pdf]

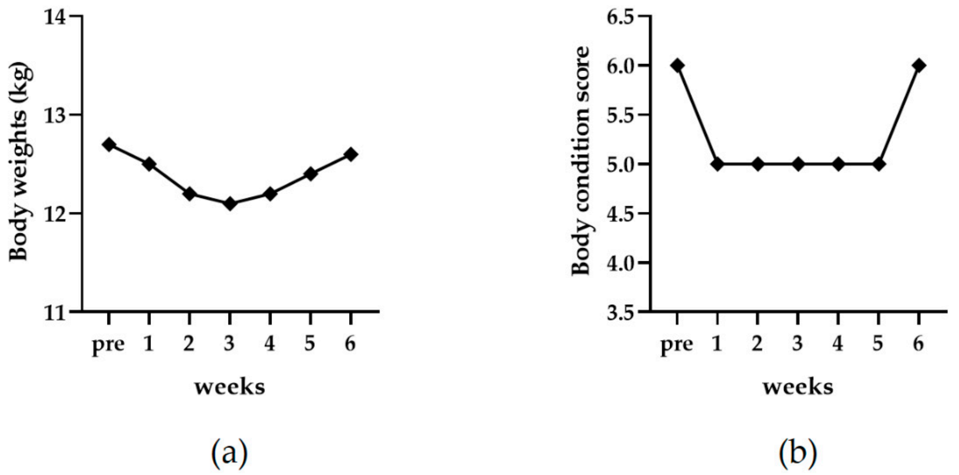

Supplementary Figure S1. Body weights and body condition scores (BCS) in separated dog. Change of body weights (a) and BCS (b) at 1-week intervals, including before environmental change with placing them into the cages (pre), for a total of 6 weeks in separated dog.

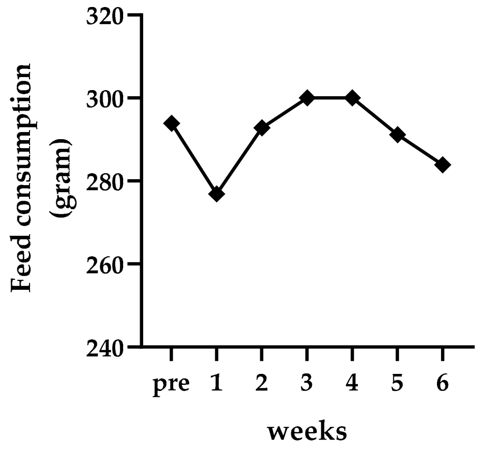

Supplementary Figure S2. Feed consumption in separated dog. Feed consumption at 1-week intervals, including before environmental change with placing them into the cages (pre), for a total of 6 weeks in separated dog.

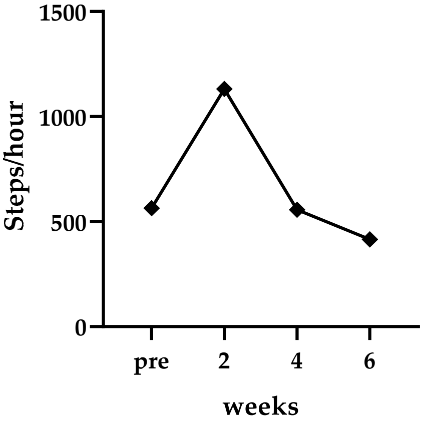

Supplementary Figure S3. Steps per hour in separated dog. Steps per hour measurement at 2-week intervals, including before environmental change with placing them into the cages (pre), for a total of 6 weeks in separated dog.

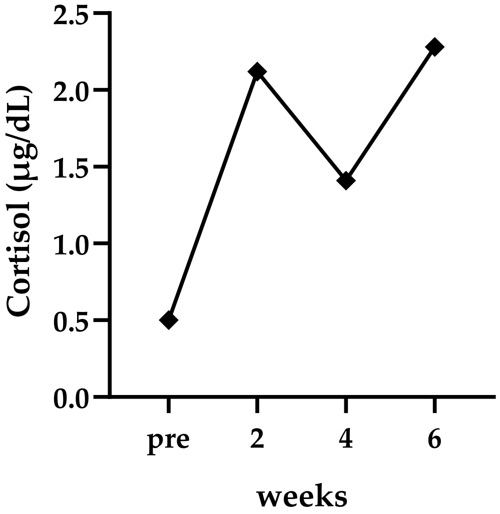

Supplementary Figure S4. Cortisol level in separated dog. Change of cortisol level in collected serum at 2-week intervals, including before environmental change with placing them into the cages (pre), for a total of 6 weeks in separated dog.

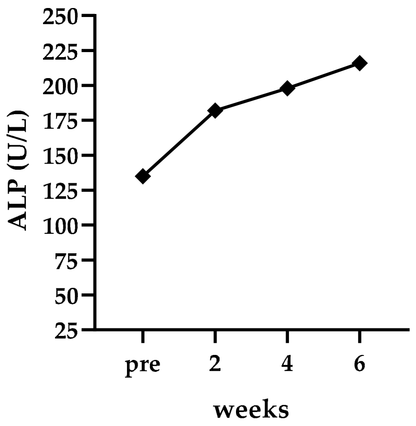

Supplementary Figure S5. Alkaline phosphatase (ALP) activity in separated dog. Change of ALP activity at 2-week intervals, including before environmental change with placing them into the cages (pre), for a total of 6 weeks in separated dog.
